# A systematic review of western medicine's understanding of pain experience, expression, assessment, and management for Australian Aboriginal and Torres Strait Islander Peoples

**DOI:** 10.1097/PR9.0000000000000764

**Published:** 2019-12-06

**Authors:** Luke Arthur, Paul Rolan

**Affiliations:** a The University of Adelaide, Adelaide Medical School, Faculty of Health and Medical Sciences, Adelaide, South Australia,; b The University of Adelaide, Faculty of Health and Medical Sciences, Adelaide, South Australia.

**Keywords:** Australia, Aboriginal and Torres Strait Islander peoples, Cultural safety

## Abstract

Although pain is a near-universal experience, pain expression and beliefs are highly variable and can affect assessment and management of pain. This systematic review seeks to synthesize the research findings regarding pain management for Australian Aboriginal and Torres Strait Islander peoples addressing variation as voiced by patients, clinicians, and researchers alike. A systematic review was performed across 7 research databases for all articles related to pain within Indigenous Australian peoples. Additional literature was identified by hand-searching reference lists. Articles were restricted to literature which addressed pain within Indigenous Australians as the primary focus of the article. Thematic analysis was performed to group articles according to those which focussed on the experience, expression, assessment, or management of pain. A total of 294 articles were identified on initial search of literature, of which 20 met inclusion criteria for this study. This review captured gross heterogeneity in cohorts, research methodologies, and conditions studied, making generalized assumptions impossible and inappropriate. Studies suggest that the beliefs of both patients and practitioners are important considerations in approaching effective assessment and management of pain. Health practitioners should appreciate how our own beliefs influence the management of patients and must ensure community consultation is undertaken in order to improve pain assessment and management.

## 1. Introduction

Pain is a near-universal experience. As a result, a significant body of research has been completed to identify optimal strategies in the quantification of pain severity and control of pain symptoms. Despite this, there is a paucity of literature regarding pain and its management for Australian Aboriginal and Torres Strait Islander peoples, from here respectfully referred to as Indigenous Australians. Of the available literature, data are derived from small cohorts with a predominant qualitative focus, where authors offer varying findings regarding the expression, assessment, and management of pain. Although some authors have suggested a range of findings regarding the effect of pain on Indigenous Australians,^6,8,9,13^ others point to short fallings in our current methods to quantify and understand pain severity and expression.^5^

From a global perspective, disparities in pain management between ethnic groups are not a unique phenomenon. A meta-analysis by Meghani et al. identified significant disparities in analgesic treatment between minority groups in the United States. In their discussion, the authors highlighted that this disparity in pain management was “sufficiently large to warrant clinical safety and quality concerns.”^17^ Despite this, little is known regarding the extent of the issue in Australia.

This systematic review intends to synthesise the research findings regarding pain management for Indigenous Australians addressing variation as voiced by patients, clinicians, and researchers alike. This will allow clinicians to reconsider their own practice and provide suggestions for researchers investigating pain management in the future.

The findings presented highlight a range of beliefs, experiences, and recommendations, which are not intended to be generalised between groups or ignore diversity within groups as suggested by current guidelines.^1,23^

## 2. Method

A systematic review was performed for literature examining pain in Indigenous Australians.

Seven databases (ATSIHealth, CINAHL, Cochrane, Embase, ERIC, PsycINFO, and PubMed) were searched on January 9, 2019, with the following search terms:

“(((Pain [MH] OR Pain* [TW] OR Analgesics [MH] OR analgesi* [TW] Or Anaes* [TW] OR anesth* OR Ache* [TW] OR Aching [TW])) AND ((Oceanic Ancestry Group [MH] OR Aborig* [TW] OR torres strait islander* [TW] OR Indigenous [TW])))) AND (Australia [MH] OR Australia* [TW] OR Queensland* [TW] OR Northern Territor* [TW] OR Victoria* [TW] OR Australian Capital Territor* [TW] OR New South Wales [TW]).”

All identified references were imported into EndNote (Clarivate Analytics 2018, x8.2) and full text retrieved. Full text was unable to be obtained for 35 references, for which abstract review identified that they did not meet inclusion/exclusion criteria or were not considered relevant to the topic. Full text of remaining documents (n = 259) were reviewed, and inclusion/exclusion criteria applied (Table 1). Of articles which met criteria (n = 85), only articles which focused on pain in Indigenous Australians (n = 20) were included. Additional articles were identified by hand-searching reference lists of articles for titles suggestive of related themes. Given the limited literature, a broad inclusion strategy was used (such as including review articles and a systematic review) as a method to increase data capture.

**Table 1:**
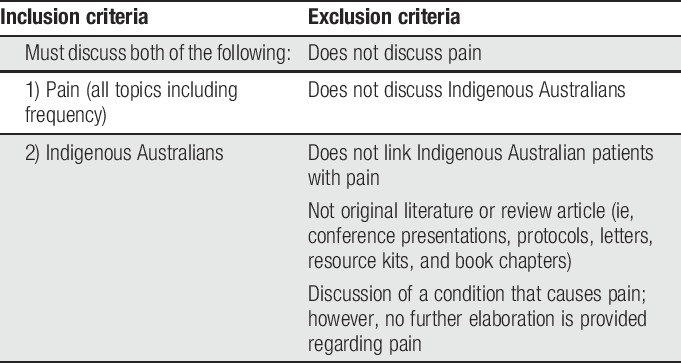
Inclusion/exclusion criteria.

All included articles were thematically assessed according to whether they discussed the themes of (1) experience, (2) expression, (3) assessment, or (4) management of pain among Indigenous Australians.

## 3. Results

**Figure 1.**
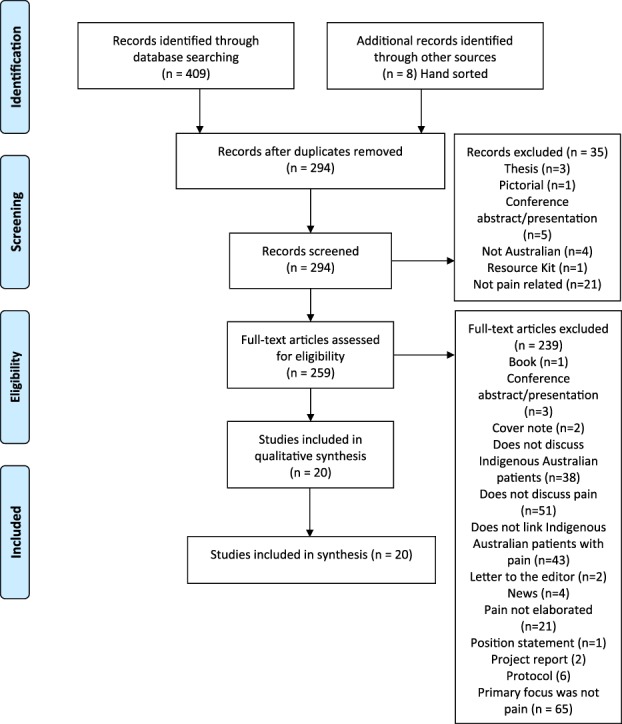
Modified PRISMA flowchart. Modified from Ref. 20.

A total of 294 unique articles were identified by database search (Fig. 1). After consideration of inclusion/exclusion criteria, 20 articles were identified which met criteria. The 20 primary articles were published between 1996 and 2018 and featured varying research methodology and geographical location of cohorts.

Of included articles, 10 were qualitative and/or descriptive studies, using interviews, surveys, or focus groups to explore the experience, expression, assessment, or management of pain in different cohorts. Six articles were cross-sectional studies, largely reporting on prevalence of pain in select populations. Other studies included a pre–post test, reports on pain assessment tools, one topic review, and one systematic review. A range of types of pain were explored by the authors, with low-back pain/musculoskeletal pain (MSP) being the most prominent (n = 10). The location of study populations varied—from South Australia (n = 1), Queensland (n = 2), New South Wales (n = 3), from Western Australia (n = 6), Northern Territory (n = 4), Central Australia (n = 3), and one national study (n = 1). Sample sizes tended to be low (12–847) and consisted of varying proportions of patients and health care providers. A range of sampling techniques were used—largely convenience or purposive sampling. With respect to authorship, 4 primary authors were responsible for 65% of the total content. This is summarised in Table 2.

**Table 2:**
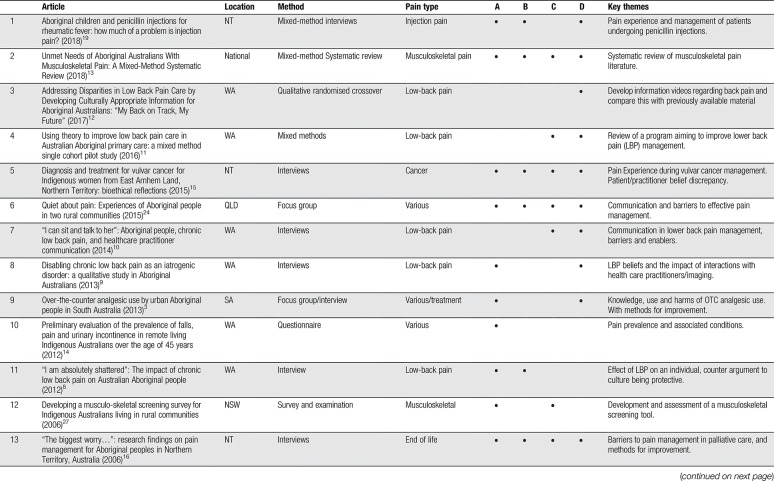

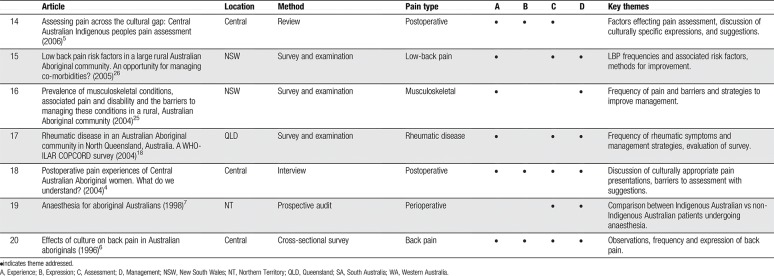
Summary and thematic analysis of included articles (n = 20).

### 3.1. Experience of pain among Indigenous Australians (n = 16)

Sixteen articles were identified that discussed the experience of pain among Indigenous Australians, with subthemes including pain beliefs, the effect of pain on an individual, and historical implications.

The first finding from the literature was the concept that patients may hold beliefs regarding the underlying cause of their pain, which differs from their practitioner. These varied from beliefs consistent with a biomedical model^9^ to beliefs such as pain being due “breaking of aboriginal law”^5^ or the “violation of taboos.”^4^ Although these findings should not be generalised beyond the cohort in which they were documented, they highlight significant variation in the beliefs held by patients or expressed by authors.

Authors also presented contrasting perspectives regarding the effect of pain on individuals. In an early article derived from the observation and interview of 56 participants, Honeyman and Jacobs^6^ found minimal “public pain or illness behavior as…recognized by the European Australian experience.” This observation was believed by the authors to be due to “community expectations about tolerating and not expressing or displaying pain.”^6^ Following this, it was stated that “these people [sic] do not regard back pain as a health issue” and “their lack of pain-related behavior and cultural views of health gives these people [sic] no motivation to consult doctors about back pain.”^6^ Contemporary authors have subsequently critiqued multiple aspects of Honeyman and Jacobs' findings, presenting contrasting views. Examples include Lin^8,9^ who suggested that variation in the associated level of disability may instead be attributed to a difference in research methodology used by authors as “a focus on cultural security may have enabled a more accurate insight.”^9^ This statement is based on the qualitative methodology used by Lin et al.^9^ who proposed that incorporating cultural security into research methodology “improves data quality and ensures that the interpretation incorporates an aboriginal cultural lens.” Although other explanations for the variation in findings have been suggested (such as “geographically and culturally different settings,”^8^ increased exposure to biomedically focused management,^9^ and culturally appropriate expressions of pain^5^), the recent critique of early articles highlights the importance of research methodology for interpretation of future research in the area. Contemporary articles continue to juxtapose the findings of the early articles and have found that chronic lower back pain could affect “multiple domains” of an individual's life,^8^ with another cohort stating, “many aboriginal people feel they need analgesia to manage everyday life.”^3^

Furthermore, the broader determinants of pain experience were discussed by 2 authors. The first being a personal communication documented by Fenwick,^5^ which stated that the discussion of pain may result in conversation regarding stolen generation and land rights. Similarly, Strong et al.^24^ conducted focus groups which noted “participants' experience of reporting physical pain is overshadowed, but not diminished, by emotional pain of many losses.” The authors state, however, that this does not suggest that “aboriginal people do not feel physical pain in the same way as nonaboriginal people.”^24^

### 3.2. Pain expression in Indigenous Australians (n = 9)

The expression of pain by Indigenous Australians was discussed in 9 articles. Included was discussion of culturally appropriate unique pain behaviours,^4,5^ such as “verbal and nonverbal silence in response to pain” as expressed by a cohort of central Australian aboriginal women.^4^ In addition, several authors identified the theme of reluctance to or not reporting pain.^6,16,24^ Theories provided by authors or their participants include males expressing “a cultural preference for bravery”^24^ and not wanting to appear weak, especially if the individual was in a leadership role.^16^ Alternatively, Strong et al. discussed participants who perceived that they were not listened to by some health professionals and stated that “a lack of established trust relationships with health care providers”^24^ may also play a role in some patients' reluctance to report pain. As an extension of pain beliefs, Fenwick and Stevens^4^ note patients may not want to discuss origins of the pain due to stigma and shame associated with certain types of pain. Another consideration was identified in Fenwick's 2006 article that documents a personal communication, which believed that “Indigenous people are reluctant to express pain due to oppression and suppression… endured since colonization.”^5^

An important consideration for clinicians, however, is Fenwick's statement (compiled from multiple references) that “Given the opportunity, indigenous people do demonstrate just as prominently and regularly unique pain behaviors and language, albeit differently from European culture.”^5^ The role of cultural expectations was also discussed by Honeyman and Jacobs when discussing illness behaviour as “recognised by European Australian experience”; before noting “if doctors, following Western practice, expected the inhabitants (sic) to actively complain and did not engage in detail enquiry, they would not discover back pain in this community.”^6^ These statements highlight how clinician expectations of pain expression may effect interpretation of observations. If extrapolated, it also highlights how research findings can be misinterpreted if research methodology does not safeguard against this.

### 3.3. Pain assessment in Indigenous Australians (n = 12)

The assessment of pain in Indigenous Australians by health practitioners was discussed by 12 articles, with themes such as communication, difficulty assessing culturally appropriate pain expression, and the use of culturally unsafe assessment methods.

A number of barriers to pain assessment were highlighted. First, Strong et al. in their 2015 article, identified participants who experienced “difficulties describing their pain problems to health professionals, in making themselves understood and in understanding what they were being told.” This was in addition to embarrassment associated with asking for clarification.^24^ Language difficulties were also noted to be a barrier to communication and hence assessment, including that associated with the use of medical jargon.^10,24^ Patient dissatisfaction could also be noted in the literature regarding patient/health care provider interactions, such as when patients received information about pain that “did not meet their expectations of an explanation and/or it contravened participants' own understandings that were based on personal experience.”^10^ Extending beyond the content which patients discussed with health care providers, Strong et al.^24^ identified patients who perceived that they were not being listen to, not being respected and believed that negative stereotypes were affecting their treatment. For the clinician, these examples highlight the importance of both implicit and explicit communication.

Variation in pain expression was also noted within the literature to impact assessment. This concept was exemplified by Fenwick and Stevens^4^ who reported that “culturally appropriate ways of expressing and managing pain are not well understood by nonaboriginal female nurses.” Extending this idea, they identified that nurses within their Central Australian cohort expected patients to “adopt pain behaviours as understood from the nurses culture”, whereas patients “expected nurses to conduct business similar to that of their own traditional … healers.”^4^ Other authors have raise concern that patients may “not be sufficiently assertive to indicate their need for pain relief”^15^ following the observation that hospitalised patients may become shy, afraid, and withdrawn.^7,15^ It should be noted that this is an incomplete list of examples sampled from the literature to highlight variation and is not intended to ignore deeper analysis as provided by the authors. Although these findings are not to be generalized outside the cohort where the finding occurred, further exploration of the authors' findings may be beneficial for clinicians working within these areas.

Cultural safety was discussed at length by Fenwick,^5^ with variations on this mentioned by 8 authors.^4,5,7–9,12,13,15^ Through this ideology, authors note that regularly used pain assessment tools and techniques may be considered unsafe in some cohorts. Examples of unsafe methods derived from a Central Australian cohort include the use of suggestive and comparative assessment methods, or using pain tools that are not significant to the patient such as 1 to 10 numerical pain scales.^5^ Despite these warnings, the development or assessment of screening tools and programs is limited to 3 tools,^18,26,27^ 1 management program,^11^ and 1 set of culturally appropriate pain information videos.^12^ In light of this, LoGiudice et al.^14^ suggested, in 2012, the need for development of “culturally appropriate assessment and management tools,” however outside the above articles, documented progress is negligible.

### 3.4. Pain management: (n = 16)

Findings regarding access to pain management were varied, with Vindigni et al.^25^ noting that 48.1% of participants “who reported experiencing pain had not accessed treatment for their musculoskeletal condition.” The reasons for not accessing treatment included “learnt to live with it” (33%), “unaware what might help” (17%), and “private therapies were too expensive” (13.2%).^25^ Similarly, Minaur et al.^18^ identified 38% of participants with rheumatic symptoms “had not sought or received any treatment”; however, when treatment was obtained, these ranged from visiting a General Practitioner (38%) to “self-treatment, with simple analgesics or tablets from friends or family” (6%). The role of family and friends in providing analgesic information and pharmaceutical sharing was also discussed in depth by Cusack et al.^3^ Among other findings, they identified that most participants had limited knowledge regarding the risks associated with over-the-counter analgesic use that when combined with an “over-reliance on information from family, friends and advertising” posed “serious health risks.”^3^ An alternate explanation regarding access to pain management was provided by a systematic review of MSP, which suggested that “lower access to care may be in part explained by qualitative experiences of care” as “aboriginal people's care experiences for MSP were predominantly negative.”^13^ This suggestion was based on articles discussing issues of cultural awareness and poor communication.

Treatment differences between Indigenous Australian and non-Indigenous Australian patients have been observed in postoperative studies. In a postoperative study by Howe et al., 1.8% (n = 5) of aboriginal patients, compared with 4.0% (n = 35) of nonaboriginal people received complex analgesia (relative risk 95% confidence interval: 0.45 [0.18–1.15]). While based on small numbers, these findings suggest a trend toward aboriginal patients receiving less complex analgesia.^7^ This is not an isolated finding, as McGrath et al.^15^ also suggests Indigenous Australian patients within their cohort “were undermedicated for pain” during vulvar cancer treatment. In addition, within a Northern Territory cohort, Mitchell et al.^19^ identified that while all clinicians within their study believed that patients found penicillin injections painful, there was inconsistent use of pain reducing measures. This was explored from a patient perspective, which found only a minority of patients in this cohort “showed the ability to negotiate about the pain of their injection with clinicians.” Of those who were empowered to negotiate, this “was linked with having a trusting relationship with clinicians.”^19^ Mixed findings were identified in Lin et al. systematic review of MSP, with multiple articles highlighting variation in treatment between Aboriginal and nonaboriginal peoples. They subsequently suggested a need to examine “the quality of health care for aboriginal people with (MSP), and if present, the determinants of care disparities.”^13^ Despite finding variation in prescribing and surgical intervention in some areas, Lin et al. also highlight data of Australian Institute of Health and Welfare, which identifies “self-reported pharmaceutical use for osteoarthritis or rheumatoid arthritis was not different between aboriginal and nonaboriginal people in nonremote areas.”^2,13^ Potential variation in treatment based on Indigenous Australian status is certainly concerning if the findings of Meghani et al.^17^ US study are extrapolated to Australia, however, whether this variation impacts outcomes remains unknown.

The impact of interactions with health care practitioners was discussed by multiple authors, with a risk of iatrogenic harm noted on multiple levels. This ranges from the suggestion of Lin et al.^9^ that “negative beliefs (held by patients regarding LBP) originated from interactions with health care practitioners suggesting disabling LBP may be partly iatrogenic.” More generally, the findings of Strong et al.^24^ suggest negative interactions with health practitioners may “deter indigenous people from seeking further services,” and a finding which was corroborated with Lin et al.^13^ in their musculoskeletal systematic review. In addition, harm may also result from the beliefs held by health care practitioners regarding pain management. An example of this was seen within a Queensland study where McGrath et al.^15^ identified that “while pain was noted as an issue by the Indigenous women,” a health practitioner in the same cohort “did not see it as an issue and indicated that indigenous peoples have high pain thresholds.”

The discussion of “pain tolerance” or “high pain threshold” has been widely critiqued including Fenwick^5^ who highlighted that if these beliefs are acted on, inadequate pain management will occur. In an earlier article, Fenwick and Stevens even stated that “the misconception that aboriginal people have a high pain tolerance requiring less pain relief is outdated and erroneous.”^4^ As recent as 2015, McGrath et al.^15^ reiterated the risk of harm in that “a cultural misunderstanding… may be occurring in which the quiet and withdrawn presentation of the indigenous women may be seen as stoicism or a high pain threshold (which) furthers women's pain and suffering.” It should be noted that our review of the literature identified no objective data examining sensory pain testing, despite beliefs of high pain threshold being held by health practitioners and focus group attendees.^15,16^ A recent study of patients receiving penicillin injections, identified a range of pain responses from neutral to “difficult to bear,” with most patients noting that “while the injections were painful, they had become resigned to them.” The author termed this response “stoic resignation” to the pain of repeated injections, however, highlighted that despite this presentation, patients “still found the experience taxing” and stressful.^19^ This reinforces an earlier caution by Fenwick in labeling a patient “stoic if they fail to vocally express their pain” as the individual may be “silent during the pain experience for a variety of reasons.”^5^ Given the lack of objective evidence to support beliefs regarding higher pain threshold/tolerance, coupled with a risk of harm, clinicians should consider how their beliefs affect patients, while researchers ought to apply caution before further propagating these views of pain tolerance through the literature.

### 3.5. Recommendations to improve assessment and management (n = 18)

The importance of communication was a frequent theme proposed to improve pain assessment and management. Suggestions included the need for improved listening to the patient by the health care provider (n = 2)^10,24^ because this was noted to result in a “greater disclosure of information” and “participants taking an active role in their management.”^10^ Communication also extended to the need for improved explanations,^24^ with the literature suggesting techniques such avoiding jargon^10,24^ and to consider the use of visual aids.^3,10,24^ The development of culturally appropriate pain education material was investigated by Lin et al. with regard to lower back pain management. Within their cohort, they identified a preference for locally developed educational material in the form of videos that involved aboriginal people. This was not, however, unanimous as some participants preferred text-based information which the author highlights as a “need for individualized patient-centred care.”^12^ Finally, alternate styles of history taking have been observed in the literature such as “yarning,” a 2-way dialogue that includes professional and social elements^10^; which is noted to assist in the development of trust.^5^ The importance of trust development is not insignificant (n = 4)^5,16,19,24^ because trust was noted as a consideration of participants before sharing vulnerabilities.^24^

The use of interpreters, liaison, and Aboriginal Health Workers was discussed by 8 articles to varying degrees.^3,5,7,10,13,18,25,26^ Benefits were noted by authors to include the provision of education to both staff and patients in addition to reducing barriers to analgesia requests.^3,7^ Howe et al.^7^ suggests the availability of a trained interpreter as minimum because, in addition to language translation, they can “convey important personal and cultural messages” to staff.

A sample of recommendations developed in specific Central Australian cohorts include Fenwick's work, which suggests the use of “verbal descriptors based on indigenous languages” or “using numerical pain assessment tools limited to the numbers 1 to 5.”^5^ Other suggestions include the importance of understanding cultural beliefs or practice.^16^

Finally, the implications of poorly managed pain is highlighted by Mitchell when discussing pain associated with penicillin injection as “the fact that it is predominantly aboriginal children requiring the injections, and the majority of clinicians who give the injections are white, and thus represent the dominant and the privileged (nonaboriginal), means that the repeated painful episodes have potential to deepen already felt inferiority and lack of power among aboriginal families.”^19^ They recommended that “a decolonizing stance would ensure that pain reduction measures are mandated,”^19^ before citing Australian guidelines for the “management of procedure-related pain in children and adolescents.”^22^

## 4. Discussion

This article captured gross heterogeneity in cohorts, research methods, and conditions studied, making generalised assumptions impossible and inappropriate. Of what is available, articles identify concerns in patient/health care provider communication, with some patients reporting dissatisfaction when they perceive that they are not being listened to, while others note not feeling respected by health professionals.^10,24^ For the practitioner, the impact of our interactions is far from inconsequential because authors identify a potential risk of iatrogenic harm after exposure to our services.^9,24^ Concern has even been raised about the cultural safety of frequently used assessment tools^5^ and practitioner behaviour,^19^ and their potential to cause a “displacement of power”^5^ away from the patient. From limited data, there is a suggestion that communication difficulties are affecting which treatments are provided to patients,^7^ but whether this affects outcomes remains unknown. Finally, while patients declare that they consider pain management to be an important issue, some practitioners continue to hold onto beliefs about high pain thresholds and pain “not being an issue”^15^ potentially hindering management. Unfortunately, these views persist despite not being supported by any objective data and in the face of authors concerns that these beliefs may place the patient at risk of harm.

For an issue as quintessentially important as pain management, we must ask the question, are we failing Indigenous Australians with service we provide? Similarly, do these concerns also apply to the other minorities groups which we treat? Unfortunately, with limited data (sporadically extracted from diverse studies consisting of predominantly lower tier levels of evidence), our ability to quantify the extent of this issue, let alone consider improving its management, is limited. This is not a new issue, however, as authors have previously stated the need for improved services and research, but little has been documented regarding our progress.

A limitation of this study was our decision to not elaborate on nuances found in specific cohorts such as cultural rules and interactions. This should not, however, distract from the importance of the findings of individual authors because these specific findings may be beneficial for practitioners working within these populations. Another limitation of this study was the decision to exclude documents outside the inclusion criteria such as resource kits,^21^ theses, and articles where pain was not a primary focus. Given the small pool of published research, the opportunity for critique and peer review was considered of utmost importance, evaluation that may have been limited in gray literature, hence our decision to exclude these resources. In addition, this document reflects the importance of research methodology, therefore including data from authors who have incidentally noted findings in Indigenous Australians while studying their own research questions may lack the research methodology required to assume validity of results. Despite including key literature, a risk of bias exists due to the inclusion of qualitative literature derived from small and diverse cohorts. While our findings are useful for the population where they were identified, we acknowledge significant risk if the findings are generalised beyond the original cohort without further research. In addition, estimates put forward by authors on prevalence/incidence of pain and its management may be influenced by recall bias or selection bias and may underestimate or overestimate true frequency of findings in these populations, and as such, generalisation should be performed with caution.

Extending from this, research methodology has certainly evolved over the period of this study, with more recent articles incorporating concepts of cultural safety, consultation, and engagement with Indigenous Australians throughout all stages of the research process. This is more consistent with guidelines produced by organisations such as Wardliparingga Aboriginal Research Unit (South Australian Health and Medical Research Institute) in the South Australian Aboriginal Health Research Accord document.^23^ The impact, which research methodology has on result validity (as reflected by critiques of past articles), highlights the importance of future research being performed under such guidelines. The principles identified in publications such as the South Australian Aboriginal Health Research Accord cannot be overstated for future research because it is through genuine consultation and adherence to these guidelines that we can collaboratively consider translational improvements to pain management.

## 5. Conclusion

Although pain is a near-universal experience, pain expression and beliefs are highly variable, variability which is noted to affect assessment and management of pain. As a result, we have presented general suggestions for improving pain management in addition to cohort-specific findings.

Through appreciating how our own beliefs affect management of our patients and deliberate community consultation, we have the potential to be a benchmark for improving pain assessment and subsequent management in not just Indigenous Australians, but potentially minority and disadvantaged groups globally.

## Disclosures

L. Arthur reports nonfinancial support from Australian and New Zealand College of Anaesthetists (ANZCA)/Faculty of Pain Medicine (FPM), other from Australian and New Zealand College of Anaesthetists (ANZCA)/Faculty of Pain Medicine (FPM), during the conduct of the study. P. Rolan reports personal fees from Bionomics, other from Lipotek, grants and personal fees from Novartis, personal fees from Seqirus, other from GPN Vaccines, other from Lateral Pharma, personal fees and other from Havah Therapeutics, outside the submitted work.

This work was originally funded through a University of Adelaide, School of Medical Sciences Summer Research Scholarship (2011).

This work has never been published; however, this article is an extension of the presentation that was awarded the Faculty of Pain Medicine Best Free Paper Award at the 2015 Australia and New Zealand College of Anaesthetists Annual Scientific Meeting (ANZCA ASM) and later presented at a session entitled “Pain and Culture” at the 2016 ANZCA ASM.
